# Impact of Asthma on the Severity of Serious Pneumococcal Disease

**DOI:** 10.4172/2161-1165.S3-001

**Published:** 2011-12-25

**Authors:** Ravneet K Dhillon, Barbara P Yawn, Kwang Ha Yoo, Thomas G. Boyce, Robert M Jacobson, Michaela E McGree, Amy L Weaver, Young J Juhn

**Affiliations:** 1 Department of Pediatric and Adolescent Medicine, Mayo Clinic, Rochester, MN; 2 Department of Research, Olmsted Medical Center, Rochester, MN; 3 Department of Internal Medicine, Konkuk University College of Medicine, Seoul, Korea; 4 Department of Health Sciences Research, Mayo Clinic, Rochester, MN

**Keywords:** Asthma, Epidemiology, Pneumococcal disease, Atopy, Severity, Prognosis, Pneumonia, Invasive pneumococcal disease, Vaccine, Serious pneumococcal disease

## Abstract

We recently reported an increased risk of serious pneumococcal disease (SPD) in asthmatics. Little is known about the impact of asthma status on the severity of SPD. We compared the severity of serious pneumococcal disease (SPD) between patients with asthma and those without asthma. The study subjects were Rochester, Minnesota residents who developed SPD between 1964 and 1983. SPD and asthma status were ascertained by using explicit predetermined criteria Severity of SPD was assessed using intensive care unit (ICU) admission rate and total days of ICU stay and hospitalization associated with treatment for SPD. We found that there were no significant differences in severity outcomes between asthmatics (n=11) and non-asthmatics (n=163). Asthma status may increase the risk of SPD but not influence its severity. However, given a small sample size of our study, a larger study needs to be considered to clarify the relationship between asthma and severity of SPD.

## Introduction

A previous study assessed underlying conditions among patients aged 2–64 years who developed invasive pneumococcal diseases (n=3,469) before the introduction of hepavalent Pneumococcal Conjugate Vaccine (PCV-7) [1]. Only 50.6% (n=1,755) had at least one condition that was a known indication for either the pneumococcal polysaccharide or conjugate vaccine [1]. Thus, a significant proportion of patients who developed Invasive Pneumococcal Disease (IPD) did not have the high-risk conditions. Talbot et al. [2] assessed whether asthma is associated with the risk of IPD and they reported that having a diagnosis of asthma is associated with an increased risk of IPD, (odds ratio (OR): 2.4, 95% Confidence Interval (CI): 1.9–3.1) in their study population who received Medicaid insurance. Subsequently, Juhn et al. [3] reported that asthmatics had a higher risk of Serious Pneumococcal Disease (SPD) including IPD and/or pneumococcal pneumonia during the primarily pre-pneumococcal vaccine era, 1964–1983. As a result, the Advisory Committee on Immunization Practices (ACIP) now recommends a single dose of 23-valent Pneumococcal Polysaccharide Vaccine to adults (19–64 years of age) [4].

Given the increased risk of development of Serious Pneumococcal Disease (SPD) in individuals with asthma, whether asthma status influences severity of SPD may be an important question to be addressed because severity and risk of infection can have different risk factors and mechanisms. Addressing this question may be important in determining the prognosis and management of SPD when asthmatics acquire SPD. Currently, there is no population-based study that examined the relationship between asthma and the severity of SPD. To determine the association between asthma status and severity of SPD, we conducted a retrospective cohort study. In this study, we assessed the impact of asthma status on the severity of SPD in the same study population during the primarily pre-pneumococcal vaccine era, 1964–1983 who was studied for the association between asthma and the risk of SPD.

## Methods

The study was approved by both the Institutional Review Boards at Mayo Clinic and Olmsted Medical Center. This is a population-based retrospective cohort study designed to assess whether asthma status is associated with the severity of SPD among the Rochester residents who developed SPD between 1964 and 1983, a primarily pre-pneumococcal vaccine era. SPD cases were identified through reviewing 3,941 medical records and asthma status was ascertained by predetermined criteria for asthma (Table 1).

### Study Design and Setting

The study setting and population were previously described in detail [3]. Briefly, Rochester, Minnesota is an excellent setting to conduct a retrospective study because medical care is virtually self-contained within the community and the Rochester Epidemiology Project provides information on all Rochester residents who had received medical care from one of two primary medical centers in Rochester. All diagnostic information has been indexed since 1935 using Berkson codes even before International Classification of Diseases (ICD) codes were available [5]. The incidence rate of asthma in Rochester was 238 per 100,000, which is comparable to those in other communities such as Tecumseh, Michigan (250/100,000) [6].

### Study Subjects

We reported the details for identification of SPD cases previously [3]. Briefly, a total of 85 different medical index search codes (Berkson codes and ICD codes) were used to identify potential SPD cases and each potential case was then confirmed by medical records review. We reviewed medical records of all 3,941 persons and identified 174 SPD cases during the period 1964 to 1983. Case definition of SPD included IPD cases (isolation of *Streptococcus pneumoniae* from a normally sterile site such as blood or cerebrospinal fluid), and/or pneumococcal pneumonia requiring all three of the following criteria, 1) a physician diagnosis of pneumonia, 2) the isolation of pneumococcus from sputum gram-stain or culture, and 3) the documented pneumonia by chest radiograph. We defined the index date of onset of the SPD as the date of documented isolation of *S. pneumoniae.*

### Ascertainment of asthma status

After all SPD cases were identified and confirmed, we used the previously collected data on asthma during the period 1964 to 1983 to ascertain the asthma status among the confirmed SPD cases (Table 1) [7]. In the original study, random samples of records were reviewed by different nurse abstractors and analyzed for inter-observer reliability and agreement rates between abstractors and a high degree of concordance was found [8]. A number of publications on asthma research used these criteria to define asthma [9–18].

### The Severity of SPD

We used the number of days of inpatient hospitalization due to SPD as the primary surrogate measure for severity of SPD. A previous study showed that the number of days of inpatient hospitalization was well correlated with severity of IPD [19]. We defined the number of days of inpatient hospitalization for SPD as days spent on all inpatient floors (including intensive care units) from admission associated with subsequent diagnosis of SPD to discharge. In addition, the severity of SPD was measured using the intensive care unit (ICU) admission rate, days of ICU stay, and a total duration of outpatient and inpatient antibiotic therapy for SPD. Patients who were readmitted to the hospital had their length of stay calculated by adding the duration of the two hospitalizations together.

### Data analysis

Socio-demographic and clinical characteristics of SPD cases with and without asthma were descriptively summarized (Table 2). The total days of ICU stay, hospitalization, and antibiotic therapy, respectively, were compared between the two groups (asthmatics vs. non-asthmatics) using the Wilcox on rank sum test. Also, the percentage of patients admitted to the ICU was compared between the two groups (asthmatics vs. non-asthmatics) using the Fisher’s ex act test. All calculated p-values were two-sided and p-values less than 0.05 were considered statistically significant.

## Results

### Study Cohort

A total of 3,941 records were reviewed and we identified 174 SPD cases. Of the 174 SPD cases, 11 cases (6.3%) were asthmatics. Socio-demographic and clinical characteristics of SPD cases with and without asthma are summarized in Table 2.

There were no significant differences in SPD severity between asthmatics and non-asthmatics (Table 3). The percentage of patients admitted to the ICU was 27.3% and 16.0% for asthmatics and non-asthmatics, respectively (p=0.40). The mean days of ICU stay were 0.9 ± 2.1 days in asthmatics (median=0) compared to 0.7 ± 2.5 in non-asthmatics (median=0, p=0.32). The mean days of hospitalization for asthmatics were 10.3 ± 8.9 days (median=7) whereas those for non-asthmatics were 9.7 ± 9.6 days (median=7, p=0.72).

## Discussion

Our previous study showed an increased risk of SPD in asthmatics. However, the current study results suggest that there were no significant differences in all outcome measures for severity of SPD between asthmatics and non-asthmatics. The literature on IPD suggests that persons with more severe illness, such as meningitis, had longer duration of hospitalization compared with those who had less severe illness such as bacteremia or pneumonia [19]. Laurichesse et al. [19] reported that in adult patients with IPD, the mean duration of hospitalization was 10.6 days (range 1–56 days). They also reported that the duration of hospitalization ranged from 4.4 ± 4.7 days for patients with bacteremia without complication to 11.5 ± 9.5 days for those with sepsis with pneumonia and 18.5 ± 9.5 for those with meningitis. Therefore, our study results indicate that the duration of hospitalization for SPD in adult subjects (9.8 days with the range of 0–62 days, n=153) are similar to that (10.6 days) reported by Laurichesse et al. [19], although these results need be compared cautiously since we assessed SPD while the previous study included only IPD. At any rate, according to our data, asthma status has little or no influence on the severity of SPD, although no data is available to compare our study results on the hospital duration for SPD in asthmatics.

Why does asthma status have a different impact on the incidence and severity of SPD? We can only postulate it. The literature suggests asthmatics have Th2-predominant biological milieu [20] and have suboptimal or impaired innate and adaptive immunity against microbial agents [21–29] including pneumococci specifically [30]. Th2-cytokines (e.g., IL-4 or IL-5) have a reciprocal counter-regulatory relationship with Th1-cytokines (e.g., IFN-gamma) [31]. Thus, suboptimal immunity in asthmatics may be associated with the increased risk of SPD but Th2 cytokines may be beneficial in down-regulating Th1-cytokines or their related pro-inflammatory mediators, which may result in excessive inflammatory response leading to tissue damages or complications such as acute respiratory distress syndrome (ARDS) or severe inflammatory response syndrome (SIRS) due to immune dysregulation during SPD [32,33]. Thus, asthma status as a Th2-predominant immune condition may not necessarily result in more severe SPD. Whether this stipulation is true needs to be further studied.

The strengths of the study include population-based study design during the primarily pre-pneumococcal vaccine era, self-contained health care system with unified medical record system for research, and independent ascertainment of SPD cases and asthma status. Also, there are inherent limitations in the study due to its retrospective design. We did not measure any laboratory or clinical parameters for severity of SPD. Although the hospital duration is a commonly used indicator for severity, future study needs to include laboratory or clinical measures for severity of SPD. Our study had a small sample size so that the study findings may be subject to type-2 error. Our study subjects were predominantly Caucasian population and it is cautious to generalize our study findings to other study settings.

In conclusion, although asthma status has been reported to be associated with the increased risk of SPD, it does not influence the severity of SPD. Given a small sample size of our study, a study with a larger sample size needs to be considered to clarify the relationship between asthma and severity of SPD.

## Figures and Tables

**Table 1:** Definition of Asthma.

Patients were considered to have definite asthma if a physician had made a diagnosis of asthma and/or if each of the following three conditions were present, and they were considered to have probable asthma if only the first two conditions were present: History of cough, dyspnea, and/or wheezing, OR history of cough and/or dyspnea plus wheezing on examination, Substantial variability in symptoms from time to time or periods of weeks or more when symptoms were absent, and Two or more of the following: Sleep disturbance by nocturnal cough and wheeze Nonsmoker (14 years or older) Nasal polyps Blood eosinophilia higher than 300/uL Positive wheal and flare skin tests OR elevated serum IgE History of hay fever or infantile eczema OR cough, dyspnea, and wheezing regularly on exposure to an antigen Pulmonary function tests showing one FEV1 or FVC less than 70% predicted and another with at least 20% improvement to an FEV1 of higher70% predicted OR methacholine challenge test showing 20% or greater decrease in FEV1 Favorable clinical response to bronchodilator Patients were excluded from the study if any of these conditions were present: Pulmonary function tests that showed FEV1 to be consistently below 50% predicted or diminished diffusion capacity Tracheobronchial foreign body at or about the incidence date Hypogammaglobulinemia (IgG less than 2.0 mg/mL) or other immunodeficiency disorder Wheezing occurring only in response to anesthesia or medications The following diseases excluded the patient from study if they occurred before the incidence date: Bullous emphysema or pulmonary fibrosis on chest radiograph PiZZ alpha1-antitrypsin Cystic fibrosis Other major chest disease such as juvenile kyphoscoliosis or bronchiectasis FVC forced vital capacity; FEV1, forced expiratory volume in 1 sec.

**Table 2:** Socio-demographic and Clinical Characteristics of SPD with and without asthma.

	No asthma n=163	Asthma n=11
**Age at index date**		
Mean (SD)	56.9 (26.7)	57.6 (24.3)
**Gender**		
Male	82 (50.3%)	6 (54.5%)
Female	81 (49.7%)	5 (45.5%)
**Ethnicity**		
Hispanic/Latino	1 (0.6%)	0 (0%)
Asian	2 (1.2%)	0 (0%)
White	153 (93.9%)	11 (100%)
Unknown	7 (4.3%)	0 (0%)
**Education**		
<High school	34 (20.9%)	1 (9.1%)
High school graduate	30 (18.4%)	2 (18.2%)
Some college	11 (6.7%)	0 (0%)
College graduate	18 (11.0%)	2 (18.2%)
Unknown	70 (42.9%)	6 (54.5%)
**Tobacco smoke exposure at index date**		
No	50 (30.7%)	4 (36.4%)
Active	51 (31.3%)	3 (27.3%)
Passive	13 (8.0%)	2 (18.2%)
Unknown	49 (30.1%)	2 (18.2%)
High-risk conditions* **for SPD prior to the index date**		
No	114 (69.9%)	9 (81.8%)
Yes	49 (30.1%)	2 (18.2%)

* High-risk conditions were based on the ACIP recommended pneumococcal vaccine-eligible conditions.

**Table 3:** Comparison between the Variables for SPD Severity in Asthmatics and Non-asthmatics.

	Asthmatics (n=11)	Non-asthmatics (n=163)	p value

ICU admission, n (%)	3 (27.3%)	26 (16.0%)	0.40

Days of ICU stay			
Mean ± SD	0.9 ± 2.1	0.7 ± 2.5	0.32
Median (IQR)	0 (0, 1)	0 (0, 0)	

Days of hospitalization			
Mean ± SD	10.3 ± 8.9	9.7 ± 9.6	0.72
Median (IQR)	7 (2, 18)	7 (3, 13)	

Days of antibiotic therapy			
Mean ± SD	13.2 ± 10.3	15.7 ± 13.5	0.61
Median (IQR)	14 (5, 16)	13 (9, 17)	

IQR, Interquantile range, 25^th^ and 75^th^ percentiles.

## Abbreviations

IPD: Invasive Pneumococcal Disease
SPD: Serious Pneumococcal Disease
95%CI: 95% Confidence Interval
ACIP: Advisory Committee on Immunization Practices
PCV-7: heptavalent Pneumococcal Conjugate Vaccine
PPV-23: 23-valent Pneumococcal Polysaccharide Vaccine
ICD: International Classification of Diseases
RR: Risk Ratio
